# ASSESSING THE IMPLEMENTATION OF COVID-19 INFECTION CONTROL BEST PRACTICES IN NURSING HOMES

**DOI:** 10.1093/geroni/igad104.2024

**Published:** 2023-12-21

**Authors:** Emily Heilbrunn, Gail D’Souza, Erica Francis, Jennifer Kraschnewski, Liza Behrens, William Calo

**Affiliations:** Penn State College of Medicine, Hershey, Pennsylvania, United States; Penn State College of Medicine, Hershey, Pennsylvania, United States; Penn State College of Medicine, Hershey, Pennsylvania, United States; Penn State College of Medicine, Hershey, Pennsylvania, United States; Penn State Ross and Carol Nese College of Nursing, State College, Pennsylvania, United States; Penn State College of Medicine, Hershey, Pennsylvania, United States

## Abstract

The COVID-19 pandemic has claimed over a million lives in the United States, with nursing homes suffering over 158,000 deaths through 2022. In a cluster randomized controlled trial (RCT) with 136 nursing homes, we delivered best-practice training to improve COVID-19 infection control practices. We sought to assess the implementation of infection control practices in participating nursing homes using Project ECHO (Extension for Community Health Outcomes) as an implementation strategy. We delivered a 16-week infectious disease and quality improvement training via real-time, interactive videoconferencing. We surveyed nursing home administrators (NHAs) and nursing staff at baseline and 6-month follow-up. Using validated items from the Centers for Disease Control and Prevention (CDC), the surveys inquired about 80 infection control best-practice activities (yes/no) related to preparedness, visitation policies, communication, protocols, management of deceased residents, education, staffing, personal protective equipment (PPE), and hygiene practices. We also assessed readiness for change and implementation climate in nursing homes with validated scales. We assessed changes in infection control practices and implementation scale scores between baseline and 6-month follow-up. A total of 197 unique participants from 126 nursing homes responded to our surveys. We found that visitation decisions were less likely made on a case-by-case basis by the end of 6 months (91% vs.76%, p=0.025) and an increase in informational materials developed for residents and families at the appropriate reading level (88% vs. 100%, p=0.021) was observed. We also saw changes in some individual items, e.g., an increase in delegating to non-physician staff

